# Comparison of Occlusal Parameters between Open Bite and Nonopen Bite Patients Using the T-Scan III System: A Pilot Study

**DOI:** 10.1055/s-0041-1739438

**Published:** 2022-01-11

**Authors:** Katika Chaikla, Jittima Pumklin, Thosapol Piyapattamin

**Affiliations:** 1 Department of Preventive Dentistry, Faculty of Dentistry, Naresuan University, Phitsanulok, Thailand; 2 Department of Restorative Dentistry, Faculty of Dentistry, Naresuan University, Phitsanulok, Thailand

**Keywords:** first tooth contact, occlusal force distribution, occlusion time, open bite, T-Scan

## Abstract

**Objective** To evaluate and compare the first tooth contact region, occlusion time, time to generate total force, and force distribution between open bite (OB) and non-OB (NOB) patients at the maximum intercuspation position using the T-Scan III system.

**Materials and Methods** Sixteen patients were divided into the OB and NOB groups ( *n* = 8 for each group). The T-Scan III system was used to evaluate the first tooth contact region, occlusion time, time to generate total force, and force distribution.

**Statistical Analysis** The mean patient age, overjet, overbite, occlusion time, and time to generate total force were compared between the groups by independent samples *t* -test. Relative force distributions between groups and among regions were compared by the Mann–Whitney *U-* and Kruskal–Wallis *H* -tests, respectively. A probability value of less than 5% ( *p* < 0.05) was considered significant.

**Results** Differences in the first tooth contact region between groups were observed. The molar region was the first tooth contact region in the OB group, while first tooth contact was observed in all regions in the NOB group. Neither the occlusion time nor the time to generate total force was significantly different between the groups ( *p* > 0.05). The highest force distributions were observed in the molar regions in both groups. Significant intragroup differences were found among all regions ( *p* < 0.05), except between the anterior and premolar regions in the NOB group ( *p* = 0.317). Intergroup differences in the force distributions in the anterior ( *p* = 0.000), premolar ( *p* = 0.038), and molar ( *p* = 0.007) regions were significant.

**Conclusion** Unlike in the NOB group, in which first tooth contact occurred in every region, in the OB group, first tooth contact occurred only in the molar region. Compared with those in the NOB group, the force distributions in the OB group were approximately 1.5 times higher in the molar region but were significantly lower in the anterior and premolar regions.

## Introduction

Open bite (OB) is described as the upper and lower teeth being separated when the jaw is completely closed; OB is rare in the posterior segment 1 and common in the anterior segment. 2 Anterior OB is characterized by a lack of occlusal contact in the anterior region when all teeth are in centric occlusion. 3

Several reports have suggested that patients with OB suffer from temporomandibular disorder (TMD). 4 5 6 Clinical signs and symptoms of TMD are exhibited among those with anterior OB malocclusion 7 8 9 and can be reduced after treatment of the malocclusion, 9 with improvements in occlusion, oral function, and esthetics. 10

Despite controversy regarding the relationship between occlusion pattern and TMD, the pattern affecting the occlusal forces distributed to the condylar head and articular disc is affected by the occlusion pattern. Research has shown that the forces are distributed to the center of the condylar head if all teeth are occluded in the maximum intercuspation position (MIP). In cases of the unilateral occlusion of posterior teeth or the occlusion of only anterior teeth, the direction of the force distribution would be changed. Thus, it can be inferred that dental occlusion affects the direction and magnitude of forces in the temporomandibular joint (TMJ) and articular disc. 11 In addition, a unilaterally overloaded occlusal force was seen in TMD patients wearing fixed dentures with disharmonious interarch relationship. 12

Aside from the occlusal force distribution, dynamic parameters interested by researchers investigating masticatory system function include the occlusion time (OT), defined as the time from the initiation of tooth contact (at self-closure into the subject's habitual intercuspation) to static intercuspation preceding and registering less force than reached in the MIP, 13 first tooth contact during mastication, and the time to generate total force (from 1 to 100%).

First tooth contact at the commencement of dental occlusion is important to the function of the masticatory system. Among subjects with bilateral neutral occlusion, one study showed first tooth contact in posterior and anterior regions in 44 and 40% of subjects, respectively, 14 while another reported corresponding values of 18.1 and 77%, respectively. 15 To the best of our knowledge, there have been no reports on the first tooth contact region, OT, and time to generate total force in OB patients. Compared with those without TMD, patients with TMD showed occlusal forces that were distributed off center, significantly longer OTs and disclusion times, and several premature contacts. Such longer OTs and premature contacts might contribute to increased TMJ friction, elevated intraarticular pressure, and subsequently condylar displacement. 16

The occlusal contact area plays an important role during mastication, and the reduction of this area can cause a decrease in performance. 17 Human bite forces vary among tooth regions in the dental arch, with the maximum force in the first molar area. 18 19 20 Since anterior teeth in those with anterior OB do not occlude, inappropriate loads may occur in the posterior region. Additionally, the possible lack of anterior guidance during occlusion may affect the TMJ status.

Compared with other materials used to record the occlusal contact area, 21 the T-Scan III computerized occlusal analysis system (Tekscan, Massachusetts, United States) is more reliable and can reveal the patient's occlusal forces, occlusal contact areas, relative occlusal force distributions, and tooth contact order. 22

At present, there have been no studies comparing the first tooth contact region, OT, time to generate total force, and occlusal force distribution between patients with and without OB using the T-Scan III system. Hence, the aim of this study was to evaluate the first tooth contact region, OT, time to generate total force, and occlusal force distribution at the MIP in orthodontic patients with OB and those without OB using the T-Scan III system.

## Materials and Methods

This study was performed in all new patients who sought orthodontic treatment at the Naresuan University Dental Hospital between July 2020 and April 2021. The inclusion criteria were the presence of a normal skeletal bite, seven fully erupted permanent teeth (except the third molar) in each quadrant, and either at least an area of OB or no OB. Patients were excluded if they had facial deformity, dental restoration, current periodontal disease, TMD, or a history of orthodontic treatment.

Patients were divided into the OB and non-OB (NOB) groups ( *n* = 8 for each group). Those in the NOB group were chosen to match those in the OB group according to Angle's classification and Steiner's analysis of sagittal skeletal malocclusion. 23

After measurement of the width of all upper teeth using a digital caliper (Mitutoyo, Kanagawa, Japan), the data were transferred to T-Scan III software (version 9.1; Tekscan) to calculate the dental arch dimensions. Each patient's occlusion was digitally recorded using the T-Scan III system. Prior to the measurement, the patient was seated upright in a dental chair (with their Frankfort horizontal plane parallel to the floor) and instructed to practice occluding into the MIP several times with a portable mirror as a reflecting object. An appropriate sensor was then placed on the occlusal surface of the maxillary arch. For predetermination, the sensor's sensitivity was adjusted by limiting only the first three pink high force columns. During the recording procedures, all patients were asked to occlude into the MIP once for 5 seconds and to repeat this occlusion process two more times. The data from three occlusions were averaged for each patient. Data from teeth showing a percentage of contact in the program but without contact with the opposing teeth were discarded to eliminate incorrect relative occlusal forces and were not included in the force distribution calculations. This procedure was conducted to reduce error, if any, from the folded sensor.

Data of first tooth contact were obtained by selecting “first contact” in the program and by observing the first contact area with a continuous force from occluding the teeth on the “force & time” graph. First tooth contact was categorized as occurring in the anterior (canine-to-canine), premolar (first and second premolars), and molar (first and second molars) regions.

The OT (second) was obtained using the “timing table” in the program from point A (first tooth contact) to point B (last contact), while the time to generate total force (1% of total force to 100% of total force) was obtained using the “force & time” graph.

**Fig. 1:**
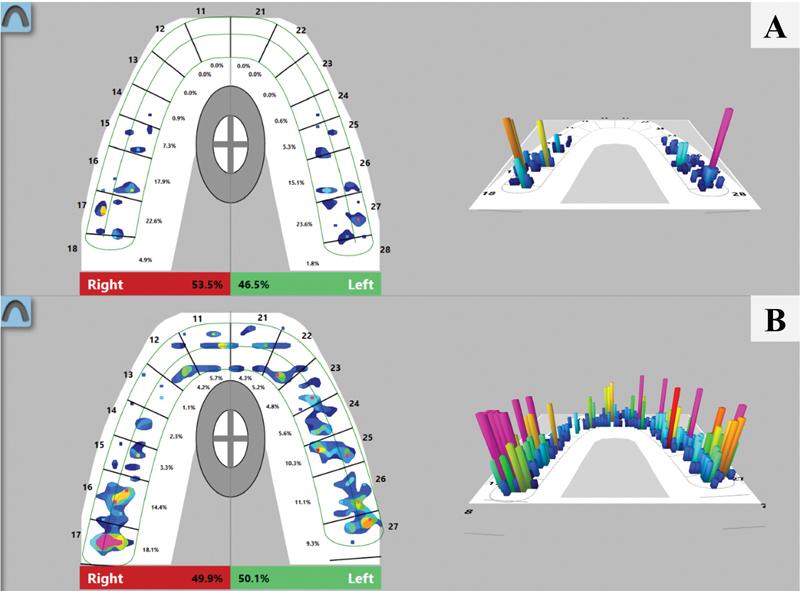
Two-dimensional (left-sided pictures) and three-dimensional (right-sided pictures) images of the occlusal force distributions in open bite **(A)** and nonopen bite **(B)** patients recorded using the T-Scan III system. All numbers adjacent to the facial side of the teeth are based on the two-digit system.

Force distributions were recorded as percentages for each tooth along the upper arch. The MIP mode was used to measure relative occlusal forces, the data of which were shown in two- and three-dimensional images ( Fig. 1 ). The relative forces in the MIP were then calculated by summation of the percentages in the anterior, premolar, and molar regions.

All numerical data were analyzed using the Statistical Package for the Social Sciences Statistics for Windows, version 23.0 (IBM; New York, United States), and the level of significance was set at *p* <0.05. The Shapiro–Wilk test was utilized to confirm the normality assumption. The mean age, overbite, overjet, OT, and time to generate total force were analyzed by independent sample *t* -test, while the mean relative occlusal force distributions were analyzed by the Mann–Whitney *U* -test. With respect to the region, the relative force distributions among the anterior, premolar, and molar regions were evaluated by the Kruskal–Wallis *H* -test, followed by post hoc comparisons.

## Results

Table 1 shows the characteristics of the patients in the OB and NOB groups. Each group contained one, two, and five patients with skeletal class I, II, and III malocclusion, respectively. Angle's class I malocclusion was observed in all patients with skeletal class I and II malocclusion and one with skeletal class III malocclusion, while Angle's class III malocclusion was observed in the remaining four patients with skeletal class III malocclusion. A significant difference ( *p* = 0.010) was detected between the groups in overjet but not age ( *p* = 1.000) or overbite ( *p* = 0.088).

**Table 1:** Details of each patient in the open bite (OB) and nonopen bite (NOB) groups ( *n* = 8 for each group)

Subject #		Skeletal type		Angle's classification		Gender		Age (y)		Overbite (mm)		Overjet (mm)		Region of first tooth contact		Occlusion time (s)		Time to generate total force (s)	
OB	NOB	OB	NOB	OB	NOB	OB	NOB	OB	NOB	OB	NOB	OB	NOB	OB	NOB	OB	NOB	OB	NOB
1	1	I	I	I	I	F	F	19	21	+2.0	+1.0	+4.5	+2.0	Mo	Mo	0.49	0.24	1.10	1.86
2	2	II	II	I	I	F	F	11	21	+3.5	+2.0	+4.0	+2.0	Mo	P	0.27	0.45	1.82	1.08
3	3	II	II	I	I	F	F	15	22	−1.0	+4.0	+4.0	+4.5	Mo	A	0.57	0.39	1.73	1.81
4	4	III	III	I	I	M	F	14	13	−1.0	+1.0	−5.0	−1.0	Mo	P	0.24	0.47	0.63	2.71
5	5	III	III	III	III	F	F	14	22	+1.5	+4.0	−3.5	−2.0	Mo	A	0.84	0.67	2.22	1.89
6	6	III	III	III	III	M	F	15	20	+1.0	+1.5	+2.0	+1.5	Mo	P	0.52	0.74	1.12	1.61
7	7	III	III	III	III	M	F	17	17	−5.0	+3.0	−4.5	+2.0	Mo	P	0.42	0.32	2.13	1.43
8	8	III	III	III	III	M	M	21	23	−1.5	+1.5	−12.0	−2.0	Mo	A	0.57	0.79	2.80	1.55
						Mean		15.75	19.88	−0.06	2.25	−1.31	0.88			0.49	0.51	1.69	1.74
						SD		3.15	3.31	2.64	1.25	5.90	2.31			0.19	0.20	0.71	0.74
						*p* -value a		1.000		0.088		0.010				0.548		0.214	

Abbreviations: A, anterior region; F, female; M, male; Mo, molar region; P, premolar region; SD, standard deviation.

a Significant difference by independent sample *t* -test at *p* <0.05.

First tooth contact in all OB patients was in the molar region, while first tooth contact occurred in all regions in NOB patients, that is, in the anterior region in three patients; premolar region, four; and molar region, one ( Table 1 ). The OT (seconds) ranged from 0.24 to 0.84 and 0.24 to 0.79 in the OB and NOB groups, respectively. No significant intergroup difference ( *p* = 0.548) in the OT was found ( Table 1 ). The time (seconds) to generate total force ranged from 0.63 to 2.80 and 1.08 to 2.71 in the OB and NOB groups, respectively. No significant difference ( *p* = 0.214) was detected between the groups in the time to generate total force ( Table 1 ).

The mean and relative occlusal force distributions in the anterior, premolar, and molar regions in each group are shown in Table 2 and Fig. 1 , respectively. In both groups, the force distributions in the molar region were highest, followed by those in the premolar and anterior regions. Significant differences ( *p* = 0.000) were detected among all regions, except between the anterior and premolar regions ( *p* = 0.38) in the NOB group ( Table 2 ).

**Table 2:** Force distributions (%) in each tooth region of the open bite and nonopen bite groups ( *n* = 8 for each group)

Force distribution (%)	Statistics	Group		*p* -Value *
		Open bite	Nonopen bite	
Anterior region	Mean	0.96 ^abc^	17.82 ^a^	0.000
	SD	2.24	9.60	
	Min	0	2.80	
	Max	6.47	29.00	
Premolar region	Mean	8.79 ^abc^	25.40 ^b^	0.038
	SD	6.50	12.51	
	Min	0	13.23	
	Max	17.23	53.60	
Molar region	Mean	89.98 ^abc^	56.13 ^ab^	0.007
	SD	8.27	11.78	
	Min	75.07	38.20	
	Max	100	73.43	
	*p* -Value **	<0.05	<0.05	

Abbreviations: Max, maximum; Min, minimum; SD, standard deviation.

Note: Similar superscript letters indicate significant intrarow differences by the Mann–Whitney *U* -test at *p* <0.05 and intracolumn differences by the Kruskal–Wallis *H* -test at *p* < 0.05.

* Mann–Whitney *U* -test.

** Kruskal–Wallis *H* -test.

Significant intergroup differences were found in all regions ( Table 2 ). The NOB group had significantly higher force distributions in the anterior ( *p* = 0.000) and premolar ( *p* = 0.0380) regions. In contrast, the OB group had a significantly higher force distribution in the molar region ( *p* = 0.007).

## Discussion

This is the first investigation into the first tooth contact region, OT, time to generate total force, and occlusal force distribution in OB and NOB patients according to the anterior, premolar, and molar regions of the dental arch.

This study strove to match patients in the two groups to eliminate confounding factors, if any, that might affect the occlusal force distribution. Only 8 out of 220 patients could be categorized into the OB group due to the presence of at least one OB area and the other inclusion criteria. NOB patients were chosen by matching them with those in the OB group according to Angle's classification of malocclusion and Steiner's classification of sagittal skeletal malocclusion. A previous report showed no significant differences in the force distribution among Angle's classes of malocclusion. 24 The mean age in the OB and NOB groups was 15.75 ± 3.15 and 19.88 ± 3.31 years, respectively, with a nonsignificant difference ( *p* = 1.000). Instead of the developmental age, the presence of seven fully erupted permanent teeth (except the third molar) in each quadrant was used as an inclusion criterion in this study. Theoretically, those teeth are fully erupted at 11.92–14.04 and 11.19–13.81 years of age in males and females, respectively. 25 Taken together, these results illustrate the usefulness of such criteria over the developmental age for future research, such as ours. There were more females than males in this study, probably reflecting their greater likelihood of seeking improvement in oral functional and orofacial esthetics, as well as their greater likelihood of attempting to receive professional dental care. 26 A significant intergroup difference ( *p* = 0.010) was found in the mean overjet value. In contrast, there was no difference in the mean overbite value between the groups ( *p* = 0.088), despite a lower mean overbite value being observed in the OB group. This might be due to the negative overbite values in 50% of the OB patients.

Our results clearly show intergroup differences in first tooth contact. First tooth contact only occurred in the molar region in the OB group. On the other hand, first tooth contact occurred in the anterior and premolar regions more frequently in the NOB group. Only one patient in the NOB group showed first tooth contact in the molar region. If the regions were divided into anterior and posterior regions, three NOB patients showed first tooth contact in the anterior region, while the remaining five NOB patients showed first tooth contact in the posterior region. A previous report demonstrated that first tooth contact was as frequent among anterior teeth as among molars. Since anterior teeth might guide masticatory function, their contact at the commencement of a chewing cycle precedes the distribution of force to posterior teeth in later stages. 14

First tooth contact during mastication plays an important role in controlling the function of jaw-elevator muscles. First tooth contact among anterior teeth, with numerous periodontal mechanoreceptors, can cause some inhibitory reflexes of the masseter and temporalis muscles. In contrast, first tooth contact among posterior teeth can result in rapidly excitatory reflexes of such muscles. 15 Regarding the occlusal forces distributed to the TMJ, first tooth contact among anterior teeth can cause increased forces in both condyles, while that among unilateral posterior teeth can cause increased pressure on the contralateral condyle. 11 15

Since first tooth contact occurred in the molar region in the OB group, some rapid excitatory reflexes of the masseter and temporalis muscles were possibly induced, causing the teeth to be occluded in the MIP. The premature contact of posterior teeth, even on one side, might also cause pressure on the contralateral TMJ and condylar displacement from increased TMJ friction, increased intraarticular pressure, and TMJ disc displacement. First tooth contact was observed in the anterior and posterior regions in the NOB group. First tooth contact in the anterior region caused less muscular activity. In this case, increased intraarticular pressure was the result of such decreased activity. Differences in the region of first tooth contact between OB and NOB patients are worth studying to determine whether OB patients are more susceptible to TMD than NOB patients. 11 15 27

Neither the OT nor the time to generate total force showed a significant difference between the groups; the mean and standard deviation OT and time to generate total force were 0.49 ± 0.19 seconds and 0.51 ± 0.20 seconds in the OB and NOB groups, respectively. These results were similar to those reported in an investigation among non-TMD subjects (0.45 ± 0.17 seconds), 28 probably because of our exclusion of TMD patients. Despite some differences in first tooth contact between our groups, there was no difference in the OT or time to generate total force ( *p* = 0.548). This illustrates that observation of only the initial and final points of occlusion might produce insufficient data. In addition, the number of patients in each of our groups was relatively small for robust data interpretation.

Some past studies have reported significant differences in the occlusal force and occlusal contact area between OB patients and controls. However, separation of the dental arch into regions has not been performed in such work among pediatric OB patients using colorimetric capsules and a spectrophotometer, 29 among adult OB patients using a surface electrode, 30 or among pediatric and adult patients with various forms of malocclusion using the Dental Prescale system. 31 Moreover, a study on the occlusal force distribution in pre- and postorthognathic surgery patients determined using the T-Scan III system divided the dental arch into three regions: the anterior (canine-to-canine) region and the left and right posterior (premolar-to-molar) regions. 32 Our results are consistent with theirs but with more details in each dental arch region.

Occlusal forces during mastication are documented to transfer from teeth to their supporting tissues. 33 A finite element analysis showed that the highest occlusal stress was at the zygomaticotemporal suture, followed by pterygomaxillary, nasofrontal, and zygomaticofrontal sutures, implying that their stress-bearing properties descended from posterior to anterior. 34 An investigation among patients wearing fixed prostheses, 18 as well as our in vivo data, showed the highest occlusal loads in the molar region. Compared with loads in the molar region in our groups, significantly smaller loads were observed in the premolar and anterior regions, while those in the anterior and premolar regions in the NOB group were similar. The phenomena can be explained by the decreased size of the respective teeth and anterior OB malocclusion in almost all of our patients. With respect to the region in the OB and NOB groups, significantly higher force distributions were observed in the anterior and premolar regions in the NOB group but in the molar region in the OB group. The latter corresponded with frequent clinical findings of anterior OB in our patients. It can be inferred that the molar region was excessively loaded, possibly resulting in some hazardous effects on the masticatory system.

Compared with healthy subjects, patients with TMD have been revealed to have a more forward position of the occlusal force center and reduced occlusal forces on the first (6.9%) and second (27%) molars. 35 In addition, those with TMJ pain showed a significantly longer distance from the occlusal force center and a significantly higher asymmetry index of the maximum occlusal load. 36 Compared with those in the NOB group, patients in the OB group had a significantly higher force in the molar region ( *p* = 0.007) but significantly lower forces in the anterior ( *p* = 0.000) and premolar ( *p* = 0.038) regions. Taken together, these results indicate that inappropriate occlusal force distributions affect the TMJ and that OB patients are prone to TMD.

There are limitations to this study. The inclusion criteria and the COVID-19 pandemic in our country have caused our sample size to be rather small. Moreover, we plan to investigate associations between skeletal malocclusion in the sagittal and vertical directions and occlusal parameters in future research.

## Conclusion

Among the selected patients in both groups with similar forms of skeletal malocclusion to reduce confounding factors, first tooth contact was observed in all three regions in the NOB group but only in the molar region in the OB group. Intergroup comparisons illustrated significant differences ( *p* < 0.05) in the occlusal force distributions in the anterior and premolar regions. In addition, the force distributions in the molar region in the OB group were approximately 1.5 times as high as those in the NOB group. There was no significant difference ( *p* > 0.05) in the OT between the groups.

## References

[1] Mitchell L An Introduction to Orthodontics 4th ed. Oxford Oxford University Press 2013 149 158

[2] Mizrahi E A review of anterior open bite Br J Orthod 1978 5 01 21 27 284793 10.1179/bjo.5.1.21

[3] Artese A Drummond S Nascimento J M Artese F Criteria for diagnosing and treating anterior open bite with stability Dental Press J Orthod 2011 16 03 136 161

[4] Henrikson T Ekberg E C Nilner M Symptoms and signs of temporomandibular disorders in girls with normal occlusion and Class II malocclusion Acta Odontol Scand 1997 55 04 229 235 9298166 10.3109/00016359709115422

[5] Egermark I Blomqvist J E Cromvik U Isaksson S Temporomandibular dysfunction in patients treated with orthodontics in combination with orthognathic surgery Eur J Orthod 2000 22 05 537 544 11105410 10.1093/ejo/22.5.537

[6] Thilander B Rubio G Pena L de Mayorga C Prevalence of temporomandibular dysfunction and its association with malocclusion in children and adolescents: an epidemiologic study related to specified stages of dental development Angle Orthod 2002 72 02 146 154 11999938 10.1043/0003-3219(2002)072<0146:POTDAI>2.0.CO;2

[7] Riolo M L Brandt D TenHave T R Associations between occlusal characteristics and signs and symptoms of TMJ dysfunction in children and young adults Am J Orthod Dentofacial Orthop 1987 92 06 467 477 3500634 10.1016/0889-5406(87)90228-9

[8] Tanne K Tanaka E Sakuda M Association between malocclusion and temporomandibular disorders in orthodontic patients before treatment J Orofac Pain 1993 7 02 156 162 8358361

[9] Kuroda S Sugawara Y Tamamura N Takano-Yamamoto T Anterior open bite with temporomandibular disorder treated with titanium screw anchorage: evaluation of morphological and functional improvement Am J Orthod Dentofacial Orthop 2007 131 04 550 560 17418724 10.1016/j.ajodo.2006.12.001

[10] Kato C Ono T Anterior open bite due to temporomandibular joint osteoarthrosis with muscle dysfunction treated with temporary anchorage devices Am J Orthod Dentofacial Orthop 2018 154 06 848 859 30477783 10.1016/j.ajodo.2017.06.030

[11] Pileicikiene G Surna A Barauskas R Surna R Basevicius A Finite element analysis of stresses in the maxillary and mandibular dental arches and TMJ articular discs during clenching into maximum intercuspation, anterior and unilateral posterior occlusion Stomatologija 2007 9 04 121 128 18303277

[12] Lila-Krasniqi Z D Shala K S Pustina-Krasniqi T Bicaj T Dula L J Guguvčevski L Differences between centric relation and maximum intercuspation as possible cause for development of temporomandibular disorder analyzed with T-scan III Eur J Dent 2015 9 04 573 579 26929698 10.4103/1305-7456.172627 PMC4745241

[13] Kerstein R B Handbook of Research on Computerized Occlusal Analysis Technology Applications in Dental Medicine 1st ed. Hershey IGI Global 2015 95 151

[14] Koos B Höller J Schille C Godt A Time-dependent analysis and representation of force distribution and occlusion contact in the masticatory cycle J Orofac Orthop 2012 73 03 204 214 22580427 10.1007/s00056-012-0075-2

[15] Vaahtoniemi L The reciprocal jaw-muscle reflexes elicited by anterior- and back-tooth-contacts-a perspective to explain the control of the masticatory muscles BDJ Open 2020 6 01 27 33335091 10.1038/s41405-020-00056-z PMC7746706

[16] Wang C Yin X Occlusal risk factors associated with temporomandibular disorders in young adults with normal occlusions Oral Surg Oral Med Oral Pathol Oral Radiol 2012 114 04 419 423 22841427 10.1016/j.oooo.2011.10.039

[17] Magalhães I B Pereira L J Marques L S Gameiro G H The influence of malocclusion on masticatory performance. A systematic review Angle Orthod 2010 80 05 981 987 20578873 10.2319/011910-33.1 PMC8939014

[18] Laurell L Lundgren D Periodontal ligament areas and occlusal forces in dentitions restored with cross-arch unilateral posterior two-unit cantilever bridges J Clin Periodontol 1986 13 01 33 38 3511102 10.1111/j.1600-051x.1986.tb01411.x

[19] Bakke M Bite force and occlusion Semin Orthod 2006 12 02 120 126

[20] Bates J F Stafford G D Harrison A Masticatory function-a review of the literature: (II) speed of movement of the mandible, rate of chewing and forces developed in chewing J Oral Rehabil 1975 2 04 349 361 23150912 10.1111/j.1365-2842.1975.tb01535.x

[21] Sharma A Rahul G R Poduval S T Shetty K Gupta B Rajora V History of materials used for recording static and dynamic occlusal contact marks: a literature review J Clin Exp Dent 2013 5 01 e48 e53 24455051 10.4317/jced.50680 PMC3892230

[22] Kim J H Computerized occlusion using T-Scan III [eBook] 2016 1 46. Accessed May 25, 2021 at:https://www.tscan.nl/wp-content/uploads/2016/12/DTL-T-Scan-Clinical-eBook.pdf

[23] Atit M B Deshmukh S V Rahalkar J Subramanian V Naik C Darda M Mean values of Steiner, Tweed, Ricketts and McNamara analysis in Maratha ethnic population: a cephalometric study APOS Trends Orthod 2013 3 05 137 151

[24] Chutchalermpan T Pumklin J Tansalarak R Sirijaroenpun S Sedtasuppana A Piyapattamin T Occlusal force distributions in various Angle's malocclusions: an evaluation by T-Scan III system J Int Dent Med Res 2019 12 02 628 632

[25] Hägg U Taranger J Dental development, dental age and tooth counts Angle Orthod 1985 55 02 93 107 3860029 10.1043/0003-3219(1985)055<0093:DDDAAT>2.0.CO;2

[26] Buunk-Werkhoven Y AB Buunk A P Fear of social rejection and oral self-care in men versus women Int Dent J 2015 65(S1):13

[27] Kerstein R B Radke J Masseter and temporalis excursive hyperactivity decreased by measured anterior guidance development Cranio 2012 30 04 243 254 23156965 10.1179/crn.2012.038

[28] Baldini A Nota A Cozza P The association between occlusion time and temporomandibular disorders J Electromyogr Kinesiol 2015 25 01 151 154 25218790 10.1016/j.jelekin.2014.08.007

[29] Corrêa E C Maeda F A de Miranda A LR Carvalho P EG da Silva L H Torres F C Masticatory evaluation of anterior open bite malocclusion using the colorimetric capsule method Gen Dent 2018 66 06 56 59 30444708

[30] Bakke M Michler L Temporalis and masseter muscle activity in patients with anterior open bite and craniomandibular disorders Scand J Dent Res 1991 99 03 219 228 1871532 10.1111/j.1600-0722.1991.tb01888.x

[31] Yoon H R Choi Y J Kim K H Chung C Comparisons of occlusal force according to occlusal relationship, skeletal pattern, age and gender in Koreans Korean J Orthod 2010 40 05 304 313

[32] Agbaje J O Casteele E V Salem A S Assessment of occlusion with the T-Scan system in patients undergoing orthognathic surgery Sci Rep 2017 7 01 5356 28706294 10.1038/s41598-017-05788-x PMC5509719

[33] Nanci A Ten Cate's Oral Histology: Development, Structure, and Function 9th ed. St. Louis Elsevier 2018 289 309

[34] Choi D S Cha B K Jang I Kang K H Kim S C Three-dimensional finite element analysis of occlusal stress distribution in the human skull with premolar extraction Angle Orthod 2013 83 02 204 211 22860753 10.2319/020112-89.1 PMC8793639

[35] Ferrato G Falisi G Ierardo G Polimeni A Di Paolo C Digital evaluation of occlusal forces: comparison between healthy subjects and TMD patients Ann Stomatol (Roma) 2017 8 02 79 88 29276576 10.11138/ads/2017.8.2.089 PMC5726858

[36] Dzingutė A Pileičikienė G Baltrušaitytė A Skirbutis G Evaluation of the relationship between the occlusion parameters and symptoms of the temporomandibular joint disorder Acta Med Litu 2017 24 03 167 175 29217971 10.6001/actamedica.v24i3.3551 PMC5709056

